# Structural and dynamic properties that govern the stability of an engineered fibronectin type III domain

**DOI:** 10.1093/protein/gzv002

**Published:** 2015-02-16

**Authors:** Benjamin T. Porebski, Adrian A. Nickson, David E. Hoke, Morag R. Hunter, Liguang Zhu, Sheena McGowan, Geoffrey I. Webb, Ashley M. Buckle

**Affiliations:** 1 Department of Biochemistry and Molecular Biology, Faculty of Medicine, School of Biomedical Sciences, Monash University, Clayton, VIC 3800, Australia; 2 Department of Chemistry, University of Cambridge, Lensfield Road, Cambridge CB2 1EW, UK; 3 Centre for Brain Research and Department of Pharmacology and Clinical Pharmacology, Faculty of Medical and Health Sciences, University of Auckland, Auckland, New Zealand; 4 Faculty of Information Technology, Monash University, Clayton, VIC 3800, Australia

**Keywords:** consensus design, fibronectin type III, FN3, molecular dynamics, stability

## Abstract

Consensus protein design is a rapid and reliable technique for the improvement of protein stability, which relies on the use of homologous protein sequences. To enhance the stability of a fibronectin type III (FN3) domain, consensus design was employed using an alignment of 2123 sequences. The resulting FN3 domain, *FN3con*, has unprecedented stability, with a melting temperature >100°C, a Δ*G*_D−N_ of 15.5 kcal mol^−1^ and a greatly reduced unfolding rate compared with wild-type. To determine the underlying molecular basis for stability, an X-ray crystal structure of FN3con was determined to 2.0 Å and compared with other FN3 domains of varying stabilities. The structure of FN3con reveals significantly increased salt bridge interactions that are cooperatively networked, and a highly optimized hydrophobic core. Molecular dynamics simulations of FN3con and comparison structures show the cooperative power of electrostatic and hydrophobic networks in improving FN3con stability. Taken together, our data reveal that FN3con stability does not result from a single mechanism, but rather the combination of several features and the removal of non-conserved, unfavorable interactions. The large number of sequences employed in this study has most likely enhanced the robustness of the consensus design, which is now possible due to the increased sequence availability in the post-genomic era. These studies increase our knowledge of the molecular mechanisms that govern stability and demonstrate the rising potential for enhancing stability via the consensus method.

## Introduction

There are currently several approaches employed to enhance protein stability. The rational approach to stabilization is challenging since it is difficult to predict the energetic and structural response to mutation in proteins, due to inaccuracies in predictive energy functions and the current inability to model the unfolded state (Magliery *et al.*, 2011). Much effort has been focused on stabilizing the native, folded state (‘positive design’ (Dantas *et al.*, 2003; Kuhlman, 2003; Shah *et al.*, 2007)) and also destabilizing the non-native states (‘negative design’ (Richardson and Richardson, 2002; Jin *et al.*, 2003)) via rational design and structural comparison of thermophilic proteins with their mesophilic counterparts (Russell *et al.*, 1994; Russell and Taylor, 1995; Auerbach *et al.*, 1997; Davlieva and Shamoo, 2010; Nakamura *et al.*, 2010; Guelorget *et al.*, 2011; Sundaresan *et al.*, 2012). Although much insight has been gained from these studies, both approaches require structures of the target protein and or any thermophilic orthologs, which then needs to be followed up with extensive structural and functional analysis. These challenges are further complicated by the context dependence of stabilizing mutations and tend to be applicable to only a small subset of scaffolds.

An alternative approach is to utilize statistical analysis of the entire protein fold, motif or domain of interest. This is an attractive idea based on the hypothesis that at a given position in a multiple sequence alignment (MSA) of homologous proteins, the respective consensus amino acid contributes more than average to the stability of the protein than non-consensus amino acids (Steipe *et al.*, 1994; Lehmann and Wyss, 2001; Magliery *et al.*, 2011). However, the technique is not always simple to implement. In particular, generation of MSAs is challenging, especially in poorly conserved regions, which leads to a large amount of noise. As most sites across a protein family are not conserved, the most common amino acid tends to be no better than picking a residue at random (Dantas *et al.*, 2003; Kuhlman, 2003; Shah *et al.*, 2007; Magliery *et al.*, 2011). Regardless, the efficacy of consensus design in improving protein stability has been demonstrated numerous times; with examples including antibodies (Steipe *et al.*, 1994; Richardson and Richardson, 2002; Jin *et al.*, 2003), the GroEL minichaperone (Wang *et al.*, 1999), the Abp1p SH3 domain (Maxwell and Davidson, 1998), the p53 DNA-binding domain (Nikolova *et al.*, 1998), fluorescent proteins (Dai *et al.*, 2007), a fungal phytase (Lehmann *et al.*, 2002) and recently the FN3 domain (Jacobs *et al.*, 2012). The availability of a small number of homologs (10–50 sequences) has typically limited the technique to combining a relatively small number of the most conserved residues with rational engineering approaches, as opposed to complete sequence redesign. With the recent advances in high throughput sequencing, the number of available sequences is rapidly growing.

In this study, we investigated whether the availability of a greater number of protein sequences resulting from advances in genomics could enhance the consensus approach. We selected the fibronectin type III (FN3) domain, a small β-sheet sandwich of roughly 90–100 amino acids in length, due to its ubiquitous nature across phyla (Fraser *et al.*, 2006), and its popularity as a model for protein folding and engineering studies (Clarke *et al.*, 1997; Hamill *et al.*, 1998, 2000b; Cota and Clarke, 2000; Koide *et al.*, 2001, 2012; Bloom and Calabro, 2009; Jacobs *et al.*, 2012; Gilbreth *et al.*, 2014). This paper describes the structural and biophysical characterization of *FN3con*—a consensus-derived FN3 domain having increased stability.

## Results

We constructed a consensus FN3 domain, which we call *FN3con*, using 2123 aligned FN3 sequences collected from the Prosite domain database (http://prosite.expasy.org/PDOC50853). The FN3 domains in this database are hand curated and sourced from numerous multi-domain proteins spanning mostly higher order eukaryotic organisms. The full MSA can be found as a FASTA file in Supplementary Data S1. We generated the new protein sequence using the consensus method, which selects the most frequently observed residue at each column of the sequence alignment. His-tagged FN3con and FNfn8 were expressed as a soluble, monomeric domain in *Escherichia coli*. Purification by nickel affinity chromatography and size exclusion chromatography produced a homogenous, monomeric sample of the expected molecular weight (Supplementary Fig. S1) that was further characterized by biophysical and X-ray crystallographic methods. We subsequently selected a set of well-studied FN3 domains (FNfn10, FNfn8 and TNfn3) and the consensus FN3 domains produced by Jacobs et al. (2012) (Fibcon and Tencon) for comparative analysis (sequences in Supplementary Data S2). All of our comparison domains have extensive biophysical data and X-ray crystal structures available, and measured stabilities ranging from 57 to 90°C.

### FN3con is the most stable FN3 domain reported

**Fig. 1:**
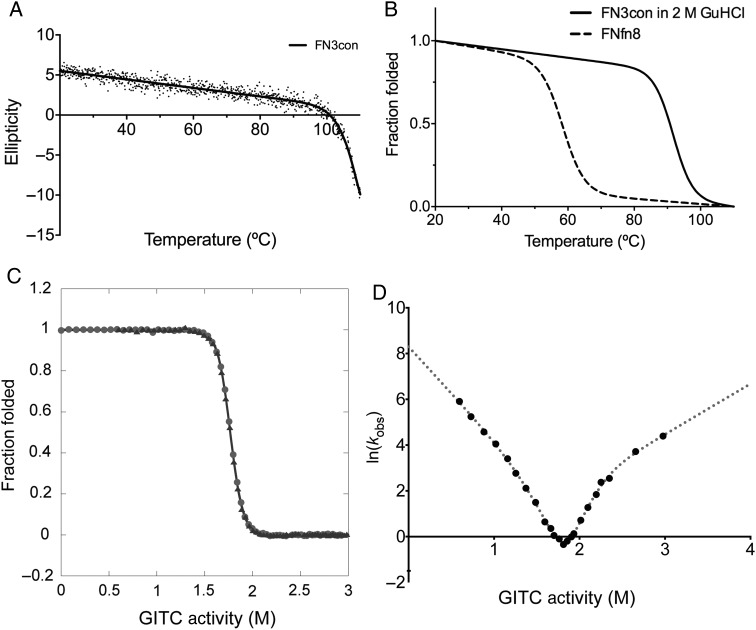
Thermal stability, chemical stability and folding kinetics of FN3con. (**A**) Thermal unfolding monitored by CD at 222 nm with non-linear fit (*R*^2^ = 0.95). (**B**) Thermal unfolding in 2 M GuHCl (solid line) and FNfn8 (dashed line) is represented as fraction folded with a non-linear fit (*R*^2^ = 0.98 and *R*^2^ = 0.76, respectively). (**C**) Equilibrium unfolding (circles) and refolding (triangles) curves. (D) Kinetic folding data showing curvature in both arms of the chevron plot.

Thermal stability of FN3con and FNfn8 was measured by circular dichroism (CD) at a wavelength of 222 nm while heating from 20 to 110°C. FN3con gradually loses secondary structure signal until ∼100°C, where a sharp unfolding transition starts but does not plateau before the thermal limit of the CD spectrophotometer is reached (110°C; Fig. 1A). We repeated the experiment in the presence of 2 M guanidine hydrochloride (GuHCl), which resulted in a complete unfolding transition and melting temperature (*T*_m_) of 90.7°C (Fig. 1B). Furthermore, we found FN3con to be reversibly foldable (Supplementary Fig. S2), a common trait of the FN3 domain (Erickson, 1994), and for comparison, we measured the *T*_m_ of FNfn8 to be 58.0°C (Fig. 1B).

The unfolding and refolding equilibrium curves of FN3con show excellent agreement with one another, further indicating that the folding is reversible (Fig. 1C). The global fit to both datasets gives a denaturant activity midpoint, [*D*′]_50_, of 1.75 ± 0.01 M, an equilibrium *m*-value, *m*_D−N_, of 8.80 ± 0.21 kcal mol^−1^ M^−1^ and hence a protein stability, Δ*G*_D−N_, of 15.5 ± 0.4 kcal mol^−1^. Note that these errors are those of the fit, and not the true errors of experimental replication. The *m*-value for FN3con (8.80 kcal mol^−1^ M^−1^) is in the range expected for homologous FN3 domains (6.38 and 9.42 kcal mol^−1^ M^−1^ for FNfn10 and TNfn3 in guanidine isothiocyanate, respectively) (Cota and Clarke, 2000). However, it is clear that FN3con is far more stable than FNfn10 and TNfn3 (15.5 compared with 9.38 and 6.68 kcal mol^−1^). The kinetic chevron (Fig. 1D) can be fitted extremely well using a modified equation to take into account both a refolding intermediate and a high-energy intermediate (see Supplementary Methods). The [*D*′]_50_ from the chevron is 1.80 ± 0.05 M, which is identical to the value obtained from the equilibrium studies and is strong evidence that both experiments are measuring the global unfolding of the protein domain and not a local effect. The fit gives a kinetic *m*-value of 10.2 ± 0.9 kcal mol^−1^ M^−1^, and a stability in buffer of 18.6 ± 1.6 kcal mol^−1^ M^−1^. Again, the errors are of the fit, and not the true errors of experimental replication. Taken together, the equilibrium and kinetic folding data indicate that, while FN3con is similar in structure to natural FN3 domains (based on the *m*-values) it is at least twice as stable. This increase in stability is predominantly due to a much slower unfolding rate, although the domain also has a slightly faster folding rate (see Table I).

**Table I.** Summary of stability, equilibrium and kinetic measurements for FN3 domains

Protein	*T*_m_ (°C)	Δ*G* (kcal mol^−1)^	Folding rate (s^−1^)	Unfolding rate (s^−1^)	Source
FN3con	>100.0	15.5 ± 0.4	4020	3.09 × 10^−8^	
Fibcon	89.6	11.4 ± 1.5	N/A	N/A	Jacobs *et al.* (2012)
FNfn10	82.5	9.38 ± 0.13	240	2.3 × 10^−4^	Cota and Clarke (2000)
Tencon	78.0	10.6 ± 0.9	N/A	N/A	Jacobs *et al.* (2012)
FNfn8	58.0	N/A	N/A	N/A	
TNfn3	57.1	6.68 ± 0.18	6.2	4.8 × 10^−4^	Hamill *et al.* (1998)

### FN3con structure reveals optimization of surface electrostatics and hydrophobic packing

**Fig. 2:**
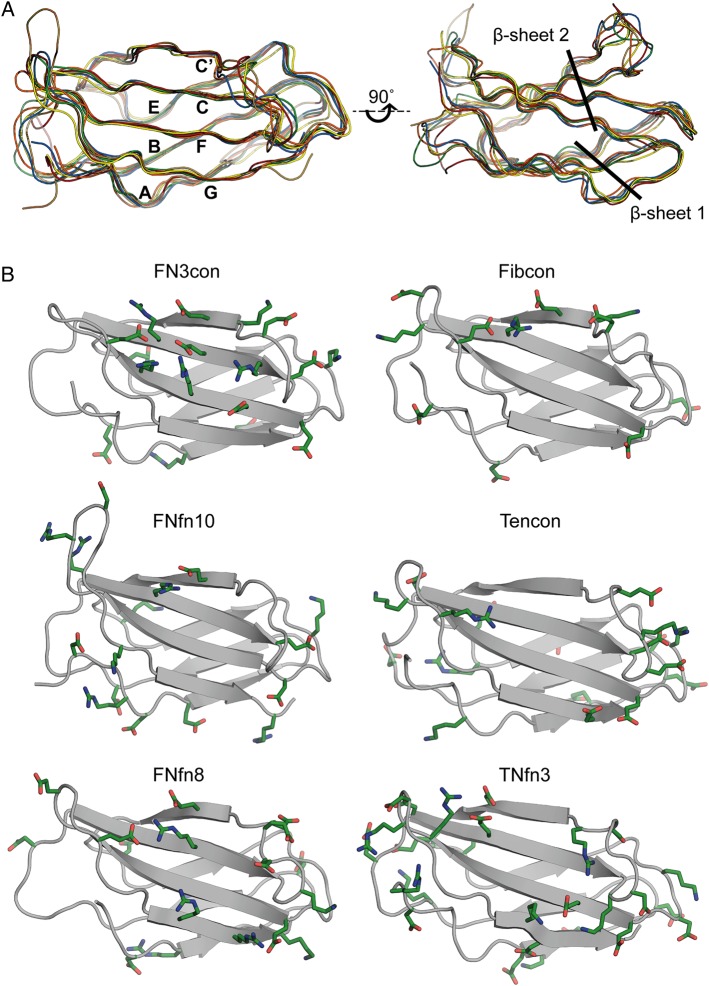
Structural alignment and distribution of charged residues on β-sheet 2 (strands C, C′, F and G). (**A**) Structural alignments of FN3con (orange), Fibcon (blue), FNfn10 (brown), Tencon (red), FNfn8 (green) and TNfn3 (yellow). (**B**) Structural analysis showing charged residues (green) of FN3con, Fibcon, FNfn10, Tencon, FNfn8 and TNfn3. Residue numbering is not included for clarity.

In order to understand the structural basis for stability in FN3con, we determined its X-ray crystal structure to 2.0 Å resolution. Data processing and structure refinement statistics are shown in Table II. FN3con adopts the FN3 fold, consisting of seven anti-parallel β-strands connected by surface exposed loops (Fig. 2). A structural alignment with our comparison domains shows very high similarity, with an average root mean square deviation (RMSD) of 1.2 Å across backbone Cα atoms in all structures (Fig. 2A).

**Table II.** Crystallographic data and refinement statistics

	FN3con (4U3H)
Data collection	
Temperature	100 K
X-ray source	Australian Synchrotron MX1
Detector	ADSC Quantum 210R
Wavelength (Å)	0.9537
Space group	*P*4_1_32
Unit cell axes (Å)	86.1, 86.1, 86.1
Angles (°)	90, 90, 90
Mol./ASU	1
Resolution (Å)^a^	35.15–1.98 (2.05–1.98)
Total reflections^a^	80 030 (95 144)
Unique reflections^a^	8078 (804)
Completeness (%)^a^	100.0 (100.0)
Multiplicity^a^	35.3 (33.5)
*R*_pim_^a^	0.018 (0.138)
〈*I*/*σI*〉^a^	28.5 (5.33)
CC1/2^a^	1.0 (0.935)
Structure refinement	
Resolution (Å)	35.15–1.98
Number of non-hydrogen atoms	801
Number of solvent molecules	61
*R*_work_ (%)	0.1970
*R*_free_ (%)^b^	0.2432
RMSD bond lengths (Å)	0.013
RMSD bond angles (°)	1.37
Ramachandran plot	
% favored (% outliers)	100.00 (0.0)
Clash score	0.7
MolProbity score	0.73 100th percentile^c^ (*n* = 12 332, 1.980 ± 0.25 Å)

a Values for highest resolution shell are in parentheses.

b The free *R* factor was calculated with 5% of data omitted from refinement.

c 100th percentile is the best among structures of comparable resolution; 0th percentile is the worst.

To investigate the structural basis for increased stability in FN3con, we first calculated several physicochemical and structural parameters that are known to affect protein stability and folding, for the set of comparison domains (Table III). Analysis reveals FN3con to have the highest number of H-bonds (46) and salt bridges (48), with the smallest accessible surface area (ASA). Comparatively, the number of H-bonds is relatively equal across the assessed domains, with a mean count of 43.5. Salt bridge counts are highly varied across the comparison set. Although FN3con has the highest number of salt bridges (48), consistent with its high stability, TNfn3 (lowest stability) has the second highest count with 41 salt bridges. However, comparisons with the ratio of acidic:basic residues show large differences between FN3con and TNfn3. Specifically, FN3con has 48 salt bridges being formed from 10 positive and 7 negatively charged residues, while TNfn3 has 41 salt bridges being formed by 18 positive and 9 negatively charged residues. Interestingly, FN3con harbors a unique and extensive complementary charged electrostatic network that is distributed over β-sheet 2, spanning strands C′, C and F. This network results from the presence of four arginine residues and four glutamic acid residues (R45, R49, R81, R83, and E47, E57, E79, E90), which are not present in any of the other FN3 domains (Fig. 2B). Comparatively, TNfn3 reveals clustering of like-charged residues on the peripheral loops (Fig. 2B).

**Table III.** Global analysis of molecular contacts

Protein	PDB code	*T*_m_ (°C)	H-bonds^a^	Salt bridge interactions <7 Å^a^	ASA (Å^2^)^b^	GRAVY score^c^	Negatively charged residues^d^	Positively charged residues^d^
FN3con	4U3H	>100	46	48	4545.5	–0.536	10	7
Fibcon	3TEU	89.6	42	14	4882.3	–0.190	8	3
FNfn10	1FNF	82.5	41	9	5470.8	–0.115	8	8
Tencon	3TES	78.0	44	28	5093.3	–0.307	12	7
FNfn8	1FNF	58.0	43	28	5239.0	–0.377	11	7
TNfn3	1TEN	57.1	45	41	5163.5	–0.577	18	9

a Hydrogen bonds and salt bridges were calculated using the WHATIF server (Hekkelman *et al.*, 2010).

b Calculated using the ASA tool from ccp4 (Winn *et al.*, 2011).

c Calculated using the ProtParam tool provided by ExPASy and uses the Kyte and Doolittle hydropathy value for each amino acid (Kyte and Doolittle, 1982).

d Negatively charged amino acids are counted as Asp and Glu, while positively charged amino acids are counted as Arg and Lys.

Calculations of ASA values correlate weakly to thermal stability, with FN3con and Fibcon having the smallest ASA values of 4545.5 and 4882.3 Å^2^ and the highest thermal stability; however, this trend does not appear to be linear for the other domains. Similarly, the grand average hydropathicity (GRAVY) scores vary quite dramatically across the set of comparison domains and are not related to thermal stability (Supplementary Fig. S3).

**Fig. 3:**
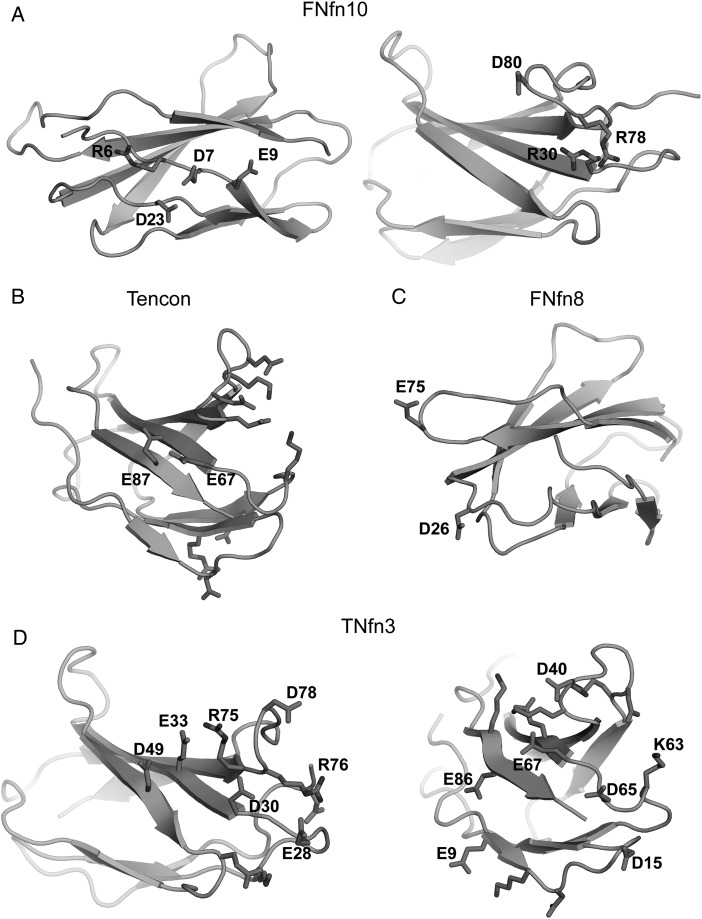
Potentially destabilizing like-charged residue clusters. (**A**) FNfn10 showing two separate clusters. (**B**) Tencon, showing E67 and E78, which are surrounded by two complementary charged clusters. (**C**) FNfn8, showing D26 and E75. (**D**) TNfn3 showing like-charged residue clusters on each set of loop hairpins. The left panel shows the N-terminal loop region, highlighting potential destabilizing interactions between E33 in strand C and D49 in strand C′, as well as a potential long range repulsion from E28, D30 and D78. The right panel shows the C-terminal loop regions, highlighting potential destabilizing interactions between E9 in strand A and E86 in strand G, D15 in the A–B loop and D65 in the E–F loop, and D40 in the C–C′ loop and E67 in strand F.

While salt bridge interactions are thought to make a relatively minor contribution to stability (Horovitz *et al.*, 1990; Serrano *et al.*, 1990), the presence of unfavorable clusters with like-charged residues is known to be destabilizing and may offer clues to the differences in stabilities of the assessed FN3 domains (Horovitz *et al.*, 1990; Loladze *et al.*, 1999; Koide *et al.*, 2001; Sanchez-Ruiz and Makhatadze, 2001). Indeed, such like-charged clusters are present in the metastable FN3 domains (FNfn10, Tencon, FNfn8 and TNfn3) but absent in the highly stable FN3con and Fibcon (Figs 2B and 3). This is clearly seen in FNfn10, which features both negatively (D7, E9 and D23) and positively (R30, R78 and D80) charged clusters (Fig. 3A). The destabilizing effect of the first cluster has been validated by mutagenesis, where mutation of D7 to asparagine or lysine increased thermal stability by ∼10°C at pH 7.0 (Koide *et al.*, 2001). Similarly, potential destabilizing clusters are also present in Tencon (E67 and E87) (Fig. 3B), FNfn8 (D26 and D52) (Fig. 3C) and TNfn3 (E33 and D49; E28, D30 and D78; E9 and E8; D15 and D65; D40 and E67) (Fig. 3D). Unsurprisingly, there is a strong similarity in the distribution of charged residues among Tencon and TNfn3. However, Tencon appears to have reduced the presence of like-charged residue clusters, resulting in increased coordination of complementary charged residues (Fig. 2).

**Fig. 4:**
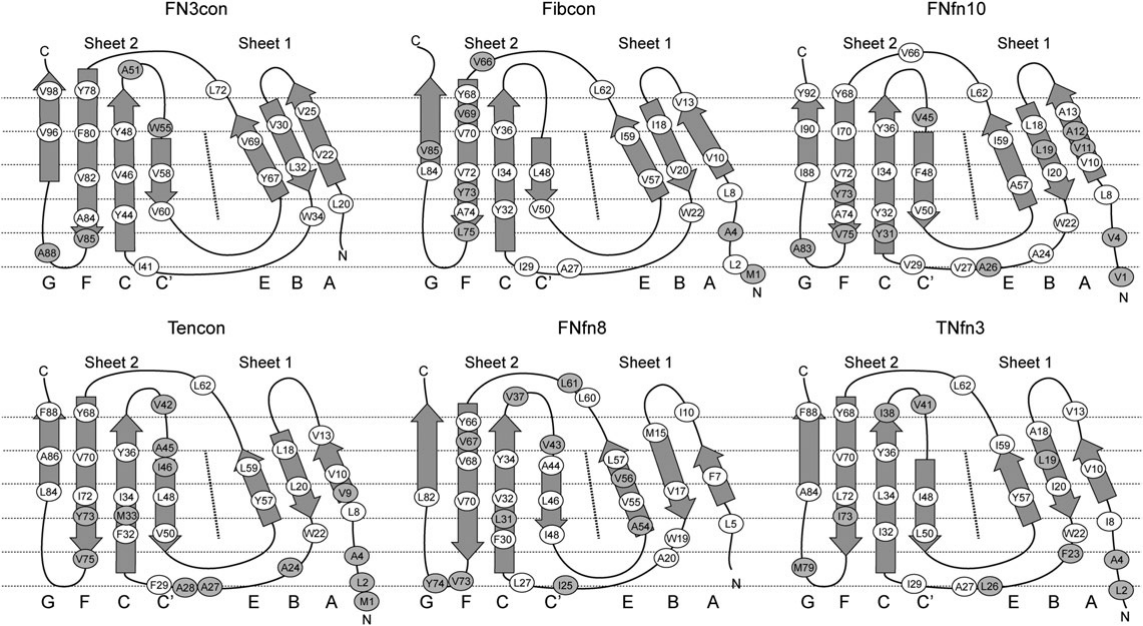
Analysis of hydrophobic residue positions in FN3 domains. A schematic unfolded model of each FN3 domain is shown, indicating positions of the hydrophobic residues as ovals. White ovals indicate the residue as contributing to the hydrophobic core and, for the most part, not solvent exposed. Shaded ovals indicate exposure to solvent and lack of contribution to the hydrophobic core.

The hydrophobic effect is a major determinant of protein folding and stability (Fersht *et al.*, 1992; Serrano *et al.*, 1992; Buckle *et al.*, 1993; Fersht and Serrano, 1993; Axe *et al.*, 1996; Kellis *et al.*, 1988). We therefore assessed differences in hydrophobic packing among the comparison set of FN3 domains, focusing on a hydrophobic ‘banding’ pattern that is orthogonal to the direction of the β-strands (Fig. 4) (Lappalainen *et al.*, 2008). Strikingly, the degree of uniformity and alignment among hydrophobic residues in each band appears to be proportional to the stability of the domain. In general, we observe higher stability to be associated with uniform hydrophobic banding as well as greater burial and reduction of bulky hydrophobic residues, which is consistent with the current understanding of the hydrophobic effect and its role in stability (Fig. 4).

As packing density of the hydrophobic core is a known factor in protein stability (Karpusas *et al.*, 1989; Chothia and Finkelstein, 1990; DeDecker *et al.*, 1996; Levitt *et al.*, 1997; Ratnaparkhi and Varadarajan, 2000), we calculated the volumes of solvent inaccessible cavities and the mean occluded surface packing (OSP) value for each FN3 domain, as a measure of packing density (Table IV). The most striking observation from these calculations is the significantly reduced solvent inaccessible cavity volume of FN3con (60.8 Å^3^) compared with the next most stable domain, Fibcon (171.0 Å^3^) (Table IV and Supplementary Fig. S4). This value alone indicates superior packing of the hydrophobic core in FN3con and may contribute to its fast folding rate. Interestingly, FNfn8 has a cavity volume of 185.8 Å^3^, suggesting that while cavity volume may be an indicator of stability, it is by no means absolute. A similar anomaly was also seen for a chimera of FNfn10 and TnFN3, which had a stability that was intermediate between the two proteins, despite having a core that was less well packed than either parent (Billings *et al.*, 2008).

**Table IV.** Packing densities of FN3 domains

Protein	*T*_m_ (°C)	Total cavity volume (Å^3^)^a^	Mean protein packing value (OSP)^b^
FN3con	>100	60.8	0.354
Fibcon	89.6	171.0	0.350
FNfn10	82.5	243.9	0.344
Tencon	78.0	260.7	0.343
FNfn8	58.0	185.8	0.356
TNfn3	57.1	334.1	0.335

a Calculated using the CASTp web server (Dundas *et al.*, 2006) with a 1.4 Å probe radius.

b Calculated using the OS software (Fleming and Richards, 2000); a higher value indicates better packing.

**Fig. 5:**
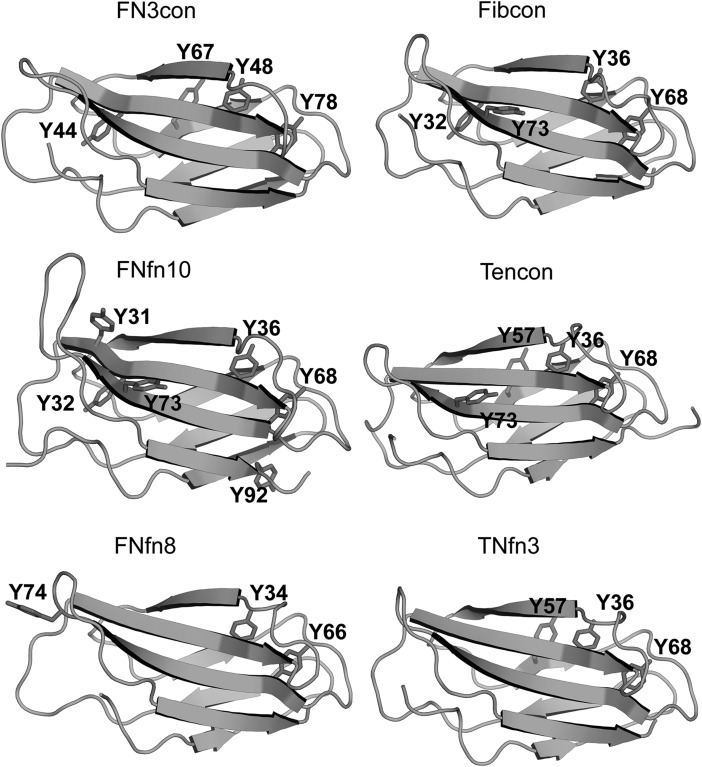
Position of tyrosine residues in FN3con, Fibcon, FNfn10, Tencon, FNfn8 and TNfn3.

We next investigated the structural context of aromatic residues, which are known to contribute greatly to the stability of immunoglobulin-like domains (Hamill *et al.*, 2000a; Nicaise *et al.*, 2003). All assessed FN3 domains contain the highly conserved tryptophan 22 (W22), while FN3con further contains a unique solvent-exposed tryptophan (W55) on β-sheet 2 (Fig. 4). W55 packs tightly against the side chains of E47, R49, E79 and R81; however, its effect on stability is not immediately apparent. Tyrosine residues are another highly conserved motif among the immunoglobulin fold and are thought to contribute to stability via the concept of a ‘tyrosine corner’, where the tyrosine residues are positioned near the beginning or end of an anti-parallel β-strand (Hamill *et al.*, 2000a; Nicaise *et al.*, 2003). Comparisons among the selected FN3 domains reveal two highly conserved tyrosine residues, one at the N-terminal end of strand C (Y48 in FN3con, Y36 or Y34 in others) and the other at the C-terminal end of strand F (Y78 in FN3con, Y68 or Y66 in others) (Figs 4 and 5). The relatively stable FN3con, Fibcon and TNfn10 contain a tyrosine residue at the C-terminal end of strand C (Y44 in FN3con and Y32 in Fibcon and FNfn10), potentially providing stabilizing interactions to both loop regions, which is absent in the less stable domains. Interestingly, FN3con, Tencon and TNfn3 share a unique tyrosine residue (Y67 and Y57, respectively) on β-sheet 1, which is absent in Fibcon, FNfn10 and FNfn8 (Figs 4 and 5).

### Simulations reveal global and local differences in the dynamics of FN3 domains

**Fig. 6:**
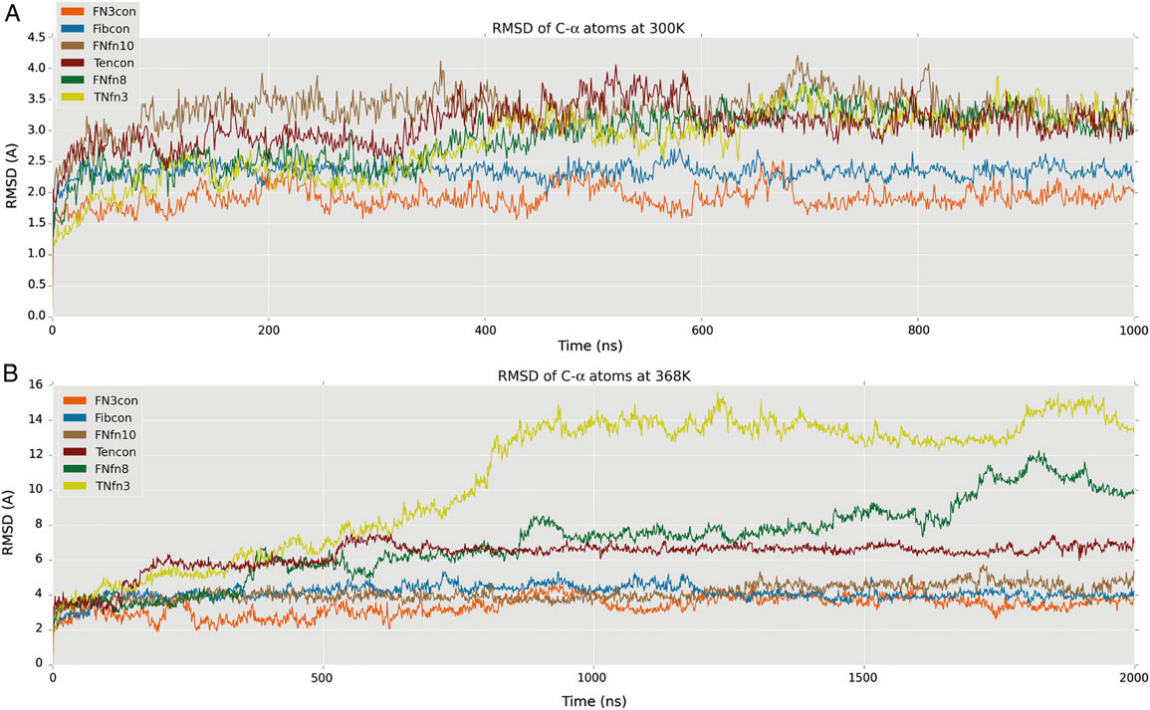
RMSD plots at 300 and 368 K. All plots represent the mean RMSD across replicate simulations (*n* = 3), for Cα atoms. (**A**) RMSD plot of FN3con (orange), Fibcon (blue), FNfn10 (brown), Tencon (red), FNfn8 (green), TNfn3 (yellow) at 300 K. (**B**) RMSD plot of FN3con (orange), Fibcon (blue), FNfn10 (brown), Tencon (red), FNfn8 (green), TNfn3 (yellow) at 368 K.

Having performed a thermodynamic, kinetic and structural characterization of FN3con we next investigated its dynamic properties. We performed MD simulations of the FN3 domains listed in Table I in triplicate at 300 K (26.9°C) for 1 μs to investigate dynamics at room temperature, and at 368 K (94.9°C) for 2 μs to investigate structural response at high temperature. All domains display a similar dynamic behavior at 300 K, showing relatively low flexibility within the β-sheet and greater motion in the flexible loops, as expected (Fig. 6A). FN3con and Fibcon are both slightly more rigid than FNfn10, Tencon, FNfn8 and TNfn3 at 300 K; however, at 368 K dramatic differences are evident. At 368 K, FN3con, Fibcon and FNfn10 remain folded, with an average RMSD of 3.4, 4.1 and 4.1 Å, respectively. Comparatively, FNfn8 and TNfn3 start to unfold after 500 ns, with unfolding essentially complete by 1 μs, while Tencon shows signs of partial unfolding in some of the replicates at ∼500 ns (Fig. 6B and Supplementary Movies S1, S2 and S3). Strikingly, the MD simulations faithfully support the experimentally derived stability hierarchy (Fig. 6B and Table I).

### Strand swapping may play a role in thermostability and unfolding

**Fig. 7:**
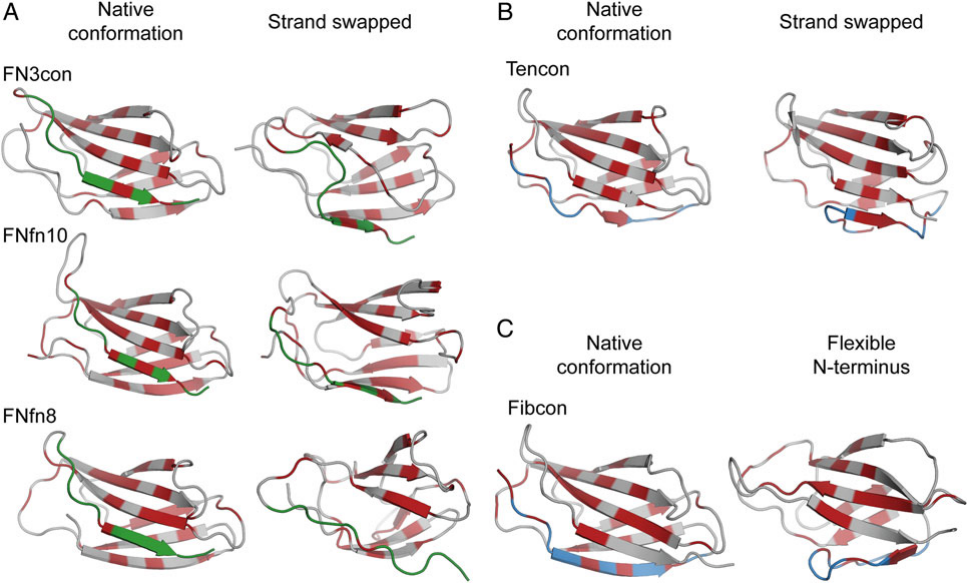
Dynamics of C- and N-terminal strands at 368 K. (**A**) Cartoon representations of FN3con, FNfn10 and FNfn8 (gray) in their native (left) and strand swapped configurations (right), showing strand G (green) and hydrophobic residues (red). (**B**) Swapping of strand A (blue) in Tencon, showing the native conformation (left) and the five-stranded β-sheet (right) and hydrophobic residues (red). (**C**) The flexible N-terminus of Fibcon, showing strand A in blue and hydrophobic residues (red).

Analysis of the simulation trajectories at 368 K reveals that, with the exception of Fibcon and TNfn3, all domains reveal some degree of strand swapping from one sheet to the other at either the N- or C-terminus. Specifically, in FN3con, FNfn10 and FNfn8, we observe strand G to swap from β-sheet 2 to β-sheet 1 (Fig. 7A and Supplementary Movie S1). Intriguingly, this is reversed in Tencon, with strand A swapping from β-sheet 1 to β-sheet 2, forming a five-stranded β-sheet (Fig. 7B and Supplementary Movie S1). The effect of strand swapping on stability is not immediately obvious from the simulations. Strand swapping at 300 K is not observed during 1 μs, which may be due to a lack of conformational sampling. Both Tencon and FNfn8 exhibit partial to full unfolding after strand swapping, suggesting that strand swapping precedes or initiates the unfolding pathway by compromising the hydrophobic core. Although no strand swapping is seen in the Fibcon simulations, we instead observe the N-terminal strand to undergo large structural rearrangements that may expose the hydrophobic core to solvent and lead to eventual unfolding (Fig. 7C and Supplementary Movie S1). In the case of TNfn3, we do not observe any strand swapping, but rather, strands A and G of TNfn3 pull closer together in concert, followed by rapid unfolding. This motion does not appear to directly initiate unfolding, which is rapid and cooperative in nature; however, it is difficult to ascertain if this is due to the simulation temperature being significantly higher than the measured melting temperature. Given the prevalent like-charged residue clusters in TNfn3, unfolding may instead be initiated by electrostatic repulsion at both peripheral loops (Fig. 3D and Supplementary Movie S2).

### The role of electrostatics in FN3 domain dynamics

Structural comparisons of FN3 domains revealed contrasting electrostatic interactions likely to induce positive or negative effects on stability (Figs 2 and 3). We therefore investigated whether electrostatics also play a role in the dynamics of FN3 domains. The complementary electrostatic mesh on β-sheet 2 of FN3con (Fig. 2) is stable throughout the simulations (at 300 and 368 K) indicating that the mesh is a stabilizing factor during the stress of high temperature, possibly by lowering the unfolding rate (Fig. 7A and Supplementary Movie S2). In contrast, one of the few surface electrostatic interactions in Fibcon (involving E47, E80 and R33) is short-lived during the simulation at 300 and 368 K and is unlikely to make a large contribution to stability (Supplementary Movie S2). During the simulations of FNfn10 at 368 K, the negatively charged cluster of D7, E9 and D23 is highly mobile, with charge repulsion causing the N-terminus to peel away into solvent, exposing the hydrophobic core. In addition, the neighboring positively charged residues R30 and R78 on strands C and F in FNfn10 rapidly rearrange throughout the simulation, with R30 burying itself into the hydrophobic core (Supplementary Movie S2). Our structural analysis of Tencon predicted charge repulsion of E67 and E87. The resulting dynamics simulations suggest that this may have some impact on the dynamics of the local area, with strands C and F regularly peeling away from one another at the E/F and C/C′ loop peripheries (Supplementary Movie S2). Finally, in FNfn8, the region surrounding residues D26 and E75 show pronounced motion prior to unfolding, suggesting a negative contribution to stability (Supplementary Movie S2).

### Rigidity of the uniform hydrophobic core of FN3 domains may contribute to their stability and folding

The hydrophobic core of FN3con is highly regular, exhibiting uniform banding of hydrophobic residues (Fig. 4). Strikingly, this uniformity is retained throughout the high-temperature simulations and after strand swapping, a phenomenon that also occurs in FNfn10 and Tencon (Supplementary Movie S1 and Fig. 7A). In particular, the uniformity of FN3con is due to residues V96 and V98 realigning with L20 and V22 in strand A as strand G swaps from β-sheet 2 to β-sheet 1 (Supplementary Movie S1).

### Dynamic recruitment of tyrosine corner residues

All of the assessed FN3 domains contain the highly conserved tyrosine residue, Y78 in FN3con (Y68/Y66 in the other domains). During the high-temperature simulations of all domains, Y78 is capable of dynamic rearrangement during strand swapping and thermal warping. Specifically, Y78 is recruited from the C′/E solvent interface to mediate solvent interactions when strand F becomes slightly separated from strand C (Supplementary Movie S3). Furthermore, the relatively stable domains of FN3con, Fibcon and FNfn10 contain a conserved tyrosine corner (residues Y44, Y32 and Y32, respectively) (Figs 4 and 5). This residue is not present in the less stable domains of Tencon, FNfn8 and TNfn3. In the simulations of FN3con, Fibcon and FNfn10, the side chains of Y44/Y32 are relatively rigid, suggesting a specialist role in stability that is consistent with other findings (Cota *et al.*, 2000). In FNfn8, a tyrosine residue is not present in this position, and as such, high-temperature simulations show that the solvent-exposed Y74 in the G/F loop is recruited to fulfill this role. However, given its position in the structure, such recruitment appears to have a destabilizing effect in the local area (Supplementary Movie S3). In Tencon, although Y73 is nearby, it is not positioned in the G/F loop, but rather at the C terminus of strand F; this positioning restricts dynamic motion and thus does not appear to play a role in stability. In TNfn3, there are no nearby tyrosine residues available to fulfill this role. FN3con contains an additional tyrosine corner motif (Y67) (Figs 4 and 5), whose interactions are almost identical to the equivalently positioned Y57 of Tencon and TNfn3, but absent in all other domains. In a previous MD simulation of TNfn3, Y36 makes several potentially stabilizing, non-crystallographic interactions (H-bonds and VdW) with Y57 and I20 (Paci *et al.*, 2003), which may indicate that the equivalent Y67 of FN3con makes a similar contribution to stability. Our simulations of FN3con show long-lived conformations of Y67 and Y48, suggesting that they play a role in stabilizing the C/E strand solvent interface (Supplementary Movie S3).

## Discussion

In this study, we have described the consensus design and subsequent biophysical, structural and dynamical characterization of a novel FN3 domain, FN3con. Design of FN3con was carried out by complete sequence generation using 2123 homologous FN3 domain sequences. In a 2012 study (Jacobs *et al.*, 2012), two consensus designed FN3 domains were described (Fibcon and Tencon). This prior work was based on using 15 FN3 repeats from human Fibronectin to generate Fibcon and 15 repeats from human Tenascin to generate Tencon, as opposed to our large non-redundant selection from the Prosite database. Their resulting consensus domains had stabilities ranging from 78°C (Tencon) to 89°C (Fibcon), and have been further stabilized to 92.7°C using alanine scanning (Jacobs *et al.*, 2012). The authors of this study suggested that the quantity of sequences influences the outcome of consensus design to a greater extent than does quality. Specifically, they showed that using 15 sequences proved superior to using 7 of the most thermostable sequences. Overall our work supports this hypothesis, in that the use of 2123 sequences during the design of FN3con has made a significant contribution to its biophysical properties. Overall, FN3con is the most stable FN3 domain reported to date, having a *T*_m_ in excess of 100°C and a Δ*G*_D−N_ of 15.5 kcal mol^−1^. It folds reversibly via two-state kinetics, with relatively fast folding and very slow unfolding rates (Fig. 1, Table I and Supplementary Fig. S2). As the FN3 superfamily is moderately conserved, with 18–41% sequence identity among the members (Fraser *et al.*, 2006), we therefore hypothesize that consensus design of a large, diverse family greatly benefits from the use of many sequences. We subsequently attribute the use of a large set of sequences to enhanced filtering of noise and a more authentic selection of conserved residues over the evolutionary landscape. In an effort to determine the molecular basis of stability in FN3con, we determined its X-ray crystal structure, which allowed structural and dynamics analyses and comparisons with Fibcon, FNfn10, Tencon, FNfn8 and TNfn3. Our results reveal that the superior stability of FN3con originates from highly specific and optimized electrostatic and hydrophobic interactions, as well as dynamic adaptability of the hydrophobic core at high temperature.

Calculations of physiochemical properties from the crystal structures revealed no relationship between the number of hydrogen bonds or salt bridges to stability. However, in a structural context, there are significant differences in the positioning of salt bridges and the ratio of positive and negatively charged residues, resulting in potential charge mismatches. The crystal structure of FN3con reveals a unique and extensive complementary charged electrostatic network that is distributed over β-sheet 2. This network consists of four arginine and four glutamic acid residues, and is not present in any of the other FN3 domains (Fig. 2B). Comparatively, TNfn3 contains a cluster of like-charged residues on the peripheral loops, which are likely to be destabilizing (Fig. 2B). The remaining FN3 domains show no sign of a linear correlation between salt bridge count and stability. This implies that stability is related to the structural context of salt bridge interactions rather than a numerical metric of potential interactions. The role of electrostatic interactions and their relation to thermal stability has been studied extensively. Surface electrostatic interactions typically make small contributions (∼0.5 kcal mol^−1^) to the overall stability, and tend to be context dependent and non-additive in nature (Serrano *et al.*, 1990). The energetic contribution provided by the electrostatic mesh in FN3con would be challenging to predict, given that each charged residue influences each other over long distances (2–7 Å) (Serrano *et al.*, 1990, 1993; Vaughan *et al.*, 2002). Although surface charged residues are unlikely to play a major role in thermodynamic stability, they may influence kinetic stability via effects on folding and unfolding rates (Cavagnero *et al.*, 1998; Karshikoff and Ladenstein, 2001; Sanchez-Ruiz, 2010). Accordingly, we hypothesize that the complementary electrostatic network seen in FN3con contributes to the dramatic reduction in unfolding rate, which has been reported for some thermophilic proteins.

Comparative analysis of hydrophobic packing in the set of FN3 domains reveals the presence of a banding pattern that is orthogonal to the direction of the β-strands (Fig. 4). This banding pattern is well known and important in formation of the folding nucleus (Lappalainen *et al.*, 2008). Strikingly, the degree of uniformity and alignment among hydrophobic residues in each band appears to be proportional to the stability of the domain. In general, we observe higher stability to be associated with uniform hydrophobic banding as well as greater burial and reduction of bulky hydrophobic residues, which is consistent with the established role of hydrophobic packing in protein stability (Fig. 4 and Table IV) (Fersht *et al.*, 1992; Serrano *et al.*, 1992; Buckle *et al.*, 1993; Fersht and Serrano, 1993; Axe *et al.*, 1996; Kellis *et al.*, 1988; Billings *et al.*, 2008). One of the most striking observations from our physiochemical properties was the dramatic decrease of solvent inaccessible cavity volume in FN3con, which is 2.8× smaller than the next best structure, Fibcon (Table IV and Supplementary Fig. S4). As packing density of the hydrophobic core is a known factor in protein stability, we suspect this attribute plays a significant role in the observed fast folding rate of FN3con (Karpusas *et al.*, 1989; Chothia and Finkelstein, 1990; DeDecker *et al.*, 1996; Levitt *et al.*, 1997; Ratnaparkhi and Varadarajan, 2000; Billings *et al.*, 2008).

Structural analysis of FN3con revealed the introduction of a cooperative electrostatic network, optimization of the hydrophobic core packing and accumulation of tyrosine corner residues in a positional pattern that is not seen in any of the other FN3 domains assessed. Given the complexity of interactions, we employed MD simulations to provide insight into the dynamics at ambient (300 K) and high temperature (368 K). Strikingly, the MD simulations at 368 K faithfully coincide with the experimentally derived stability hierarchy (Fig. 6B and Table I). Overall, the simulation trajectories reveal partial unfolding of Tencon and loss of native structure in FNfn8 and TNfn3 around 500 ns, which we attribute to the start of an unfolding pathway (Supplementary Movie S1). On closer inspection of the simulation trajectories at high temperatures, FN3con, FNfn10 and FNfn8 show C-terminal strand (strand G) swapping from β-sheet 2 to β-sheet 1 (Fig. 7A). Interestingly, as FN3con and FNfn10 strand swap the hydrophobic residues in strand G align perfectly to those in strand A (Fig. 7A and Supplementary Movie S1). This is in contrast to FNfn8, where the hydrophobic residues on strand G do not successfully align with those in strand A (Fig. 7A and Supplementary Movie S1), suggesting that the ability to realign the hydrophobic residues after strand swapping has an effect on stability. The simulations of Tencon also reveal strand swapping; however, there are dramatic differences compared with the other FN3 domains, with its N-terminal strand (strand A) swapping from β-sheet 1 to β-sheet 2 (Fig. 7B), forming a five-stranded β-sheet. Interestingly, mutations in the F/G loop of Tencon have been shown to promote strand swapping of the C-terminal strand (strand G), as well as influencing the resulting aggregation properties (Teplyakov *et al.*, 2014). However, it is unclear how this relates to the dynamics observed at 368 K, especially since strand G remains stable throughout MD of Tencon. Although there exists only one example of strand swapping within the current FN3 literature, folding studies, including Phi-value analysis, of FN3-like domains indicate folding occurs through a common-core ring involving strands B, C, E and F, leaving strands A and G to pack last (Hamill *et al.*, 1998, 2000b; Cota and Clarke, 2000). This suggests a lack of constraints on strands A and G and is consistent with the strand swapping events we observe during the high-temperature simulations (Fig. 7 and Supplementary Movie S1). We therefore hypothesize that strand swapping is an event on the unfolding pathway.

MD simulations at 368 K reveal flexibility of loop regions in all structures, providing cavities for solvent to enter and potentially destabilize the hydrophobic core. Tyrosine corners feature tyrosine residues positioned near the beginning or end of an anti-parallel β-strand. This feature is highly conserved, ubiquitous and exclusive to Greek key proteins (Hemmingsen *et al.*, 1994; Hamill *et al.*, 2000a; Nicaise *et al.*, 2003). Tyrosine corners in the FN3 superfamily are involved in early structure formation and are important for stability of the structure, with tyrosine to phenylalanine mutations costing 1.5–3 kcal mol^−1^ in stability (Hamill *et al.*, 2000a). Our analysis of tyrosine residues showed a striking trend in that the most stable FN3 domains (FN3con, Fibcon and FNfn10) all contain tyrosine corners evenly spread throughout their structures and accessible to both peripheral loop regions. Specifically, FN3con, Fibcon and FNfn10 make use of a unique tyrosine residue (Y44, Y32 and Y32, respectively) at the C-terminal end of strand C; a trait not observed in Tencon, FNfn8 and TNfn3 (Figs 4 and 5). Intriguingly, FN3con, Tencon and TNfn3 share a unique tyrosine residue (Y67, Y57 and Y57, respectively) at the C-terminal end of strand E in sheet 1 (Figs 4 and 5). It has been suggested that Y57 makes a small contribution to stability in TNfn3 by forming H-bond and Van der Waals interactions with Y36 (Paci *et al.*, 2003). It is therefore likely that this residue makes a similar contribution to the stability of FN3con, given the close similarities in its environment and the rigidity of both residues in our MD analysis. In addition, simulations at 368 K reveal the capacity for rearrangement and recruitment of tyrosine residues at high temperature. One of the most striking differences is the lack of Y44/Y32 in Tencon, FNfn8 and TNfn3. Although FNfn8 attempts to recruit the solvent-exposed Y74, which is similarly positioned to Y44/Y32, it appears to destabilize the local area (Fig. 6 and Supplementary Movie S3). Furthermore, Tencon and TNfn3 lack the ability to reposition a tyrosine residue to this region. As such, we hypothesize that the presence of a unique distribution of tyrosine corners in FN3con provides stabilizing features and may contribute to the observed slow unfolding rate.

In conclusion, we have successfully generated an FN3 domain, FN3con, which has unprecedented stability, with experimental data highlighting a *T*_m_ in excess of 100°C, a Δ*G*_D−N_ of 15.5 kcal mol^−1^, reversible folding via two-state kinetics, with the fastest folding and slowest unfolding rates reported to date. Structural and dynamical analysis reveals that FN3con stability does not result from a single mechanism, but rather the combination of several features and a strong tendency to remove non-conserved unfavorable interactions. These features include the introduction of a previously unseen complementary charged residue mesh on β-sheet 2, which we propose to contribute to the slow unfolding rate. FN3con includes the optimization of alignment within the hydrophobic core, resulting in superior packing, followed by removal of solvent-exposed hydrophobic residues and widespread adoption of tyrosine residues. Dynamics simulations reinforce the stability hierarchy determined by experiment and shed light on behavior of the FN3 domain at high temperature. Furthermore, we are the first to suggest that the flexibility and swapping of the N- and C-terminal strands of the FN3 domain are implicated in its unfolding pathway at high temperature, thus playing a role in its stability by allowing optimization of hydrophobic packing during conformational change. As such, FN3con features near perfect realignment of the hydrophobic core and recruitment of tyrosine residues. By exploiting the increased availability of genomic sequence data, this study further supports consensus design to be a rapid and effective method for the engineering of protein stability.

## Methods

See Supplementary data.

## Supplementary data

Supplementary data are available at *PEDS* online.

## Author contributions

B.T.P., G.I.W., L.Z. and A.M.B. designed the study. B.T.P. performed the protein design, expression and purification, CD thermal melt experiments, crystallography, molecular dynamics simulations and analysis. A.A.N. performed the folding kinetics and equilibrium measurement experiments. D.E.H. assisted with crystallization of FN3con. S.M. assisted with crystallography and structure determination. B.T.P. generated figures and movies with assistance from M.R.H. B.T.P., D.E.H. and A.M.B. wrote the manuscript.

## Funding

A.A.N. is supported by the Wellcome Trust (grant number WT 095195). S.M. is an Australian Research Council Future Fellow (FT100100960). G.I.W. is an Australian Research Council Discovery Outstanding Researcher Award Fellow (DP140100087). A.M.B. is a National Health and Medical Research Senior Research Fellow (1022688). Funding to pay the Open Access publication charges for this article was provided by the Australian Research Council (grant number DP150101371).

## Supplementary Material

Supplementary Data
