# Supplementary material for: Structural and dynamic properties that govern the stability of an engineered fibronectin type III domain
Source: Protein Eng Des Sel. 2015 Feb 16;28(3):67–78. doi: 10.1093/protein/gzv002 (PMC4330816; doi:10.1093/protein/gzv002)
Supplement: Supplementary Data [file supp_28_3_67__index.html]

Supplementary Data 

# Structural and dynamic properties that govern the stability of an engineered fibronectin type III domain

## Supplementary Data

Supplementary Data

**Files in this Data Supplement:**

- Supplementary Data - Doc file
- Supplementary Video 1 - mp4 file
- Supplementary Video 2 - mp4 file
- Supplementary Video 3 - mp4 file
