# Supplementary material for: Structural and dynamic properties that govern the stability of an engineered fibronectin type III domain
Source: Protein Eng Des Sel. 2015 Feb 16;28(3):67–78. doi: 10.1093/protein/gzv002 (PMC4330816; doi:10.1093/protein/gzv002)
Supplement: Supplementary Data [file supp_gzv002_gzv002supp.doc]

**SI Methods**

*Multiple Sequence Alignments*

The sequence alignment for homologous fibronectin type III domains was sourced from the Prosite database (http://prosite.expasy.org/PDOC50853). The Prosite sequence alignments are hand curated with no definitive method. The sequences were retrieved in FASTA format in February 2012, returning 2,619 entries. In order to reduce bias from the sequence alignment, the CD-HIT web server (Hekkelman et al., 2010; Winn et al., 2011) was used to remove redundant sequences above 95% similarity, reducing the sequence count to 2,123 entries. Sequences were re-aligned using ClustalW (Kyte and Doolittle, 1982; Winn et al., 2011). Application of the consensus algorithm was applied over all 2,123 sequences, resulting in a single consensus sequence for FN3con.

*Protein Expression and Purification*

Genes encoding FN3con and FnFN8, containing an N-terminal 6x HIS tag, followed by a thrombin cleavage site (LVPRGS), were chemically synthesized and provided in a pJexpress 404 plasmid by DNA2.0. The resulting plasmids were transformed into competent C41 *E. coli* cells for expression. A single colony from each transformation was picked and grown overnight at 37°C in 250 ml of 2xYT (16.0 g/L tryptone, 10.0 g/L yeast extract, 5.0 g/L NaCl) media containing 100 μg/ml of ampicillin. These cultures were then used to seed 2 L of 2xYT media for the FN3con and FNfn8. Cultures were induced at an OD_600_ of 0.9 with IPTG (0.5 mM final concentration), and grown for a further 5 hours at 37°C. The cultures were harvested and cell pellets resuspended in TBS (50 mM Tris, 150 mM NaCl, pH 7.4; EDTA free protease inhibitors, ThermoFisher), lysed via sonication and cellular debris removed by centrifugation (5,000x G). Recombinant protein was isolated from the whole cell lysate by metal affinity chromatography using loose NiNTA resin (Sigma). Protein eluted from NiNTA resin was filtered (0. 22 μm) then subjected to size exclusion chromatography using a Superdex 75 16/60 column (GE Healthcare) equilibrated in either PBS (140 mM NaCl, 2.7 mM KCl, 10 mM PO_4_^3-^, pH 7.4) for biophysical characterization or low salt TBS (50 mM Tris, 50 mM NaCl, pH 7.4) for protein crystallography. Protein concentration was determined by Nanodrop ND-1000 (ThermoFisher) and protein was stored at 4°C until use (biophysical characterization) or used immediately (protein crystallography).

*Characterisation of Thermal Stability*

Thermal stability of purified FN3con and FnFN8 was measured by monitoring the circular dichroism (CD) signal at 222 nm to assess secondary structure content. Protein samples were used at a concentration of 0.2 mg/ml. T_m_ values were measured using a Jasco J-815 CD spectrophotometer with a peltier thermal control unit (CDF-426S). A quartz cuvette with a path length of 1 mm was used throughout. Samples were heated from 20°C to 110°C at a rate of 1°C per minute and monitored at 222 nm. Far-UV scans from 195 nm and 260 nm were collected in triplicate at 20°C before and after each melt. A buffer-only scan was collected in order to calculate a baseline. Following baseline removal, data was collected in triplicate, averaged and fit to a two-state unfolding model using a non-linear least squares fitting algorithm (Dundas et al., 2006; Kyte and Doolittle, 1982). The melting temperature (*T_m_*) was calculated as the G/Mg ratio.

*Equilibrium Measurements*

A 6 M solution of guanidine isothiocyantate (GITC) in TBS was combined in varying ratios with TBS buffer using a liquid handling robot to create a range of denaturant solutions from 0 – 6 M GITC. These solutions were subsequently mixed in an 8:1 ratio with 9 µM protein in TBS to give a final concentration of 1 µM protein. All solutions were left to equilibrate at 25°C for at least three hours, after which the fluorescence of each solution was measured on a Perkin Elmer LS55 fluorimeter using an excitation wavelength of 280 nm and an emission range of 300 – 400 nm. Readings were obtained from a 1 cm pathlength cuvette maintained at 25 ± 0.1°C. The experiment was repeated, but using 9 µM protein pre-unfolded in 5 M GITC to generate a refolding curve. These solutions were left to equilibrate for at least six hours before their fluorescence was ascertained.

*Kinetic Measurements*

Folding was monitored by changes in fluorescence using a 350 nm cut-off filter and an excitation wavelength of 280 nm. All experiments were performed using an Applied Photophysics (Leatherhead, UK) stopped-flow apparatus maintained at 25 ± 0.1°C. For unfolding experiments, one volume of 11 µM protein solution was mixed rapidly with ten volumes of a concentrated GITC solution. For refolding, one volume of denatured protein in 4 M GITC solution was mixed with ten volumes of low-concentration GITC. In all cases, both solutions contained TBS buffer and were equilibrated at 25°C for at least 30 minutes before use. Data collected from at least six experiments were averaged and traces were fit to a single or double exponential function as appropriate. Due to mixing effects, data collected in the first 2.5 ms were always removed before fitting.

*Data analysis of equilibrium and kinetic measurements*

An Excel spreadsheet was used to derive the fluorescence average emission wavelength (AEW) for each of the equilibrated denaturant solutions (Dundas et al., 2006; Fleming and Richards, 2000). Excel was also used to convert each denaturant concentration into a denaturant activity since the two values are not directly proportional for GITC (Eswar et al., 2007; Fleming and Richards, 2000). A plot of AEW against denaturant activity (Kaleidagraph, Synergy Software) yielded the expected sigmoidal plot, which was fitted to the standard two-state equation (Eswar et al., 2007; Hess et al., 2008) to obtain the *m*-value (*m*_D-N_), the denaturant activity 50% ([D’]_50_) and hence the stability of the protein in TBS buffer (Δ*G*_D-N_). Both the unfolding and refolding AEW curves can be converted to Fraction Folded by first removing the baselines and then normalizing the resulting data.

All kinetic traces fitted well to a single exponential decay plus a linear drift term. Longer experiments indicated the presence of a much slower second refolding rate that was incompatible with the timescale of the stopped-flow apparatus. Since FN3con 11 proline residues, we attribute this rate to proline isomerization although further experiments are required to confirm this hypothesis. An amplitude analysis suggests that this slower rate accounts for between 50% and 80% of all proteins at low concentrations of denaturant. The resulting chevron plot (Hess et al., 2008; Oostenbrink et al., 2004) showed rollover in the refolding arm (indicating the presence of a refolding intermediate) and a kink in the unfolding arm (indicating the presence of a high energy intermediate). It was fitted using Prism (Synergy Software) to the following equation to estimate all parameters:


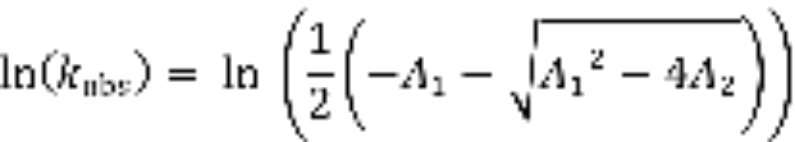


where:


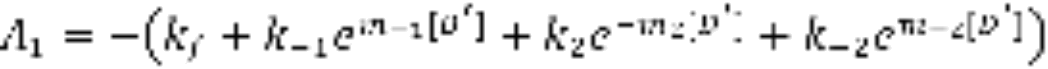


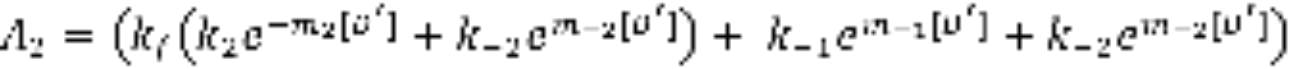


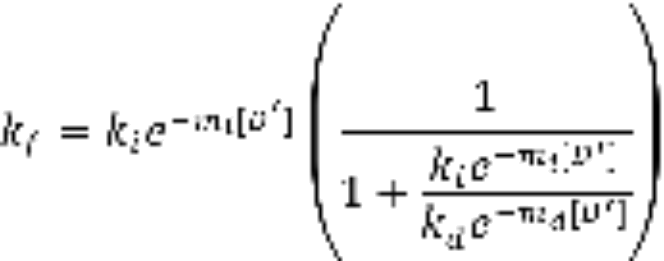


*k*_i_ and *m*_i_ are the folding rate constant from the refolding intermediate (I) to the first transition state (TS1) and its associated *m*-value, *k*_d_ and *m*_d_ are from the denatured state (D) to the first transition state (TS1), *k*_-1_ and *m*_-1_ are unfolding from the high energy intermediate (I*) over TS1, *k*_2_ and *m*_2_ are folding from the high energy intermediate (I*) over TS2, *k*_-2_ and *m*_-2_ are unfolding from the native state (N) over TS2. By convention, *k*_-1_ is set as 100,000 s^-1^ and *m*_-1_ is set as 0 M^-1^: *m*2 is thus the *m*-value between TS1 and TS2 while the ratio *k*_-1_/*k*_2_ informs on the difference in free energy between the two transition states.

*Crystallization of FN3con*

FN3con was purified in 50 mM Tris, 50 mM NaCl, pH 7.4 and was concentrated to 25 mg/ml (Milipore 3 kDa cutoff concentrator). Concentrated FN3con was filtered through a 0.22 μm centrifugal filter and crystals were obtained from 0.1M phosphate-citrate pH 4.2 and 40% PEG300 (JCSG+ Suite, Qiagen). Drops were prepared 1:1 in 1 μl. Small hexagonal or cubic crystals were formed within three days.

*X-ray diffraction, structure determination and refinement*

FN3con crystals were flash frozen in liquid nitrogen without further cryoprotection. Diffraction data was collected at the Australian Synchrotron on the MX1 beamline and initially processed with Blu-Ice (McPhillips et al., 2002). Diffraction data to 1.98 Å resolution was collected and processed with iMOSFLM (Berendsen et al., 1981; Oostenbrink et al., 2004). Complete data collection statistics can be found in Table 2. The FN3con structure was determined by molecular replacement (MR) with Phaser (McCoy et al., 2007) using PDB entry 2CK2 (Ng et al., 2007) as a search probe (following removal of solvent atoms and trimming of sidechains to create a poly-Ala model). The asymmetric unit contains one protein molecule. Model building and structure refinement was carried out with PHENIX v. 1.8.4-1496 (Adams et al., 2010) and Coot (Emsley and Cowtan, 2004). Coordinates of FN3con were deposited in the RCSB Protein Data Bank with PDB ID 4U3H.

*Structure analysis*

In analysis of FN3con, residue numbering was kept as per amino acid positions of the construct, due to non-ideal sequence to structure alignment with the other FN3 domains (sequences in Data S2). In analysis of FNfn8, residues 1238-1325 in 1FNF were renumbered 1-88 with residue P1238 as residue 1. In analysis of FNfn10, residues 1416-1509 of 1FNF were renumbered 1-94 with residue V1416 as residue 1. In analysis of TNfn3, residues 802 to 891 from the original PDB file of 1TEN were renumbered 1-90 with residue R802 as residue 1. In analysis of Fibcon and Tencon, residue numbering was unchanged and is per respective PDB files 3TEU and 3TES. C-terminal His tags from Fibcon and Tencon were removed for structural analysis and molecular dynamics simulations.

Structural alignments were performed using the Mustang-MR webserver (Konagurthu et al., 2010). H-bonds and salt-bridges (<7 Å) were calculated using the WHATIF server (Hekkelman et al., 2010). Accessible surface area (ASA) was calculated using the ASA tool from CCP4 (Hekkelman et al., 2010; Winn et al., 2011). The grand average hydropathy (GRAVY) score was calculated using the ProtParam tool provided by ExPASy and uses the Kyte and Doolittle hydropathy value for each amino acid (Kyte and Doolittle, 1982; Winn et al., 2011). Total cavity volume was calculated using the CASTp web server (Dundas et al., 2006; Kyte and Doolittle, 1982) using a 1.4 Å probe radius. Mean occluded surface packing (OSP) was calculated using the OS software (Dundas et al., 2006; Fleming and Richards, 2000).

*System setup for molecular dynamics simulations*

Simulations of FN3con, Fibcon, FNfn10, Tencon, FNfn8 and TNfn3 were based on the following crystal structures with PDB codes 4U3H, 3TEU, 1FNF, 3TES, 1FNF, 1TEN respectively. Coordinates were prepared by removal of crystal waters, N- or C-terminal His tags and extracted from their respective PDB files as per listings in the *Structure analysis* methods section. Residues with missing atoms were modelled using MODELLER (Eswar et al., 2007; Fleming and Richards, 2000), followed by capping of the N- and C-termini with the neutral *N*-methyl amide and acetyl groups. All residues were simulated at their dominant protonation state at pH 7. Completed structures were solvated in a cubical simulation box with a minimum distance of 1.4 nm from any protein atoms to the box wall, followed by the addition of sodium and chloride ions to neutralize the system. Extra NaCl was added to reach a final concentration of approximately 150 mM NaCl. System dimensions and compositions are listed in Table S1.

**Table S1.** Simulation system dimensions and composition

| System | Dimensions | Sodium ions | Chloride ions | Water molecules | Total atoms (approximate) |
| --- | --- | --- | --- | --- | --- |
| FN3con | 6.8 nm^3^ | 32 | 29 | 10,262 | 31,700 |
| Fibcon | 7.3 nm^3^ | 40 | 35 | 12,313 | 37,800 |
| FNfn10 | 7.0 nm^3^ | 31 | 31 | 10,888 | 33,600 |
| Tencon | 7.0 nm^3^ | 36 | 31 | 11,019 | 31,700 |
| FNfn8 | 7.1 nm^3^ | 15 | 11 | 11,600 | 35,700 |
| TNfn3 | 7.2 nm^3^ | 44 | 34 | 11,905 | 36,700 |

*Simulation protocol*

All simulation systems were subjected to energy minimization, followed by equilibration in the NPT ensemble (26.85 °C (300 K), 1 bar (~1 atm)) or (94.85 °C (368 K), 1 bar (~1atm)), with 1,000 kJ mol^-1^ nm^-2^ positional restraints applied to all non-hydrogen atoms; restraints were stepped down 10 fold every 100 ps over 300 ps. Equilibrated systems were run at 300 K and 368 K for 1 μs and 2 μs, in triplicate, with each replicate starting from a different distribution of initial velocities. All simulations were performed using GROMACS ver 4.0.7 (Eswar et al., 2007; Hess et al., 2008) in conjunction with the GROMOS 53A6 united-atom force field (Hess et al., 2008; Oostenbrink et al., 2004). Water was represented explicitly using the simple-point-charge (SPC) model (Berendsen et al., 1981; Oostenbrink et al., 2004). All simulation systems were performed in an NPT ensemble under periodic conditions. Temperature was maintained close to its reference value of 300 K or 368 K by V-rescale temperature coupling (Berendsen et al., 1984; McCoy et al., 2007). Pressure was maintained close to a reference value of 1 atm by isotropic coupling with a Berensden pressure bath (Berendsen et al., 1984; Ng et al., 2007). Non-bonded interactions were evaluated using a twin-range cut-off scheme: interactions falling within the 0.8 nm short-range cutoff were calculated every 2 fs whereas interactions within the 1.4 nm long cutoff were updated every 10 fs, together with the pair list. A generalized reaction-field correction was applied to the electrostatic interactions beyond the long-range cutoff (Adams et al., 2010; Tironi et al., 1995), using a relative dielectric permittivity constant of ɛRF = 62 as appropriate for SPC water (Emsley and Cowtan, 2004; Heinz et al., 2001). All bond lengths to hydrogen atoms were constrained using the P-LINCS algorithm (Hess et al., 1997; Konagurthu et al., 2010) and water geometry was constrained using the SETTLE algorithm (Hekkelman et al., 2010; Miyamoto and Kollman, 1992). A leap-frog integrator (Hess et al., 2008) was used throughout, with a time step of 2 fs.

*Simulation Analysis*

Analyses of the simulations were performed using the tools provided in the GROMACS package 4.0.7 (Hess et al., 2008) and custom scripts in conjunction with ProDy (Bakan et al., 2011). Graphs and plots were produced using Matplotlib (Hunter, 2007). Molecular graphics were prepared with PyMol ver. 1.3.2 (DeLano, 2002) and Visual Molecular Dynamics (VMD) 1.9.2 (Humphrey et al., 1996).

**References**

Adams, P.D., Afonine, P.V., Bunkóczi, G., Chen, V.B., Davis, I.W., Echols, N., Headd, J.J., Hung, L.-W., Kapral, G.J., Grosse-Kunstleve, R.W., et al. (2010). PHENIX: a comprehensive Python-based system for macromolecular structure solution. Acta Crystallogr. D Biol. Crystallogr. *66*, 213–221.

Bakan, A., Meireles, L.M., and Bahar, I. (2011). ProDy: protein dynamics inferred from theory and experiments. Bioinformatics *27*, 1575–1577.

Berendsen, H.J.C., Postma, J.P.M., van Gunsteren, W.F., DiNola, A., and Haak, J.R. (1984). Molecular dynamics with coupling to an external bath. J. Chem. Phys. *81*, 3684.

Berendsen, H., Postma, J., van Gunsteren, W.F., and Hermans, J. (1981). Interaction models for water in relation to protein hydration. Intermolecular Forces *11*, 331–342.

DeLano, W.L. (2002). The PyMOL Molecular Graphics System. (2002).

Dundas, J., Ouyang, Z., Tseng, J., Binkowski, A., Turpaz, Y., and Liang, J. (2006). CASTp: computed atlas of surface topography of proteins with structural and topographical mapping of functionally annotated residues. Nucleic Acids Res. *34*, W116–W118.

Emsley, P., and Cowtan, K. (2004). Coot: model-building tools for molecular graphics. Acta Crystallogr. D Biol. Crystallogr. *60*, 2126–2132.

Eswar, N., Webb, B., Marti-Renom, M.A., Madhusudhan, M.S., Eramian, D., Shen, M.-Y., Pieper, U., and Sali, A. (2007). Comparative protein structure modeling using MODELLER. Curr Protoc Protein Sci *Chapter 2*, Unit2.9.

Fleming, P.J., and Richards, F.M. (2000). Protein packing: dependence on protein size, secondary structure and amino acid composition. Journal of Molecular Biology *299*, 487–498.

Heinz, T.N., van Gunsteren, W.F., and Hünenberger, P.H. (2001). Comparison of four methods to compute the dielectric permittivity of liquids from molecular dynamics simulations. J. Chem. Phys. *115*, 1125.

Hekkelman, M.L., Beek, te, T.A.H., Pettifer, S.R., Thorne, D., Attwood, T.K., and Vriend, G. (2010). WIWS: a protein structure bioinformatics Web service collection. Nucleic Acids Res. *38*, W719–W723.

Hess, B., Bekker, H., Berendsen, H.J.C., and Fraaije, J.G.E.M. (1997). LINCS: a linear constraint solver for molecular simulations. J. Comput. Chem. *18*, 1463–1472.

Hess, B., Kutzner, C., van der Spoel, D., and Lindahl, E. (2008). GROMACS 4:  Algorithms for Highly Efficient, Load-Balanced, and Scalable Molecular Simulation. J. Chem. Theory Comput. *4*, 435–447.

Humphrey, W., Dalke, A., and Schulten, K. (1996). VMD: visual molecular dynamics. J Mol Graph *14*, 33–8–27–8.

Hunter, J.D. (2007). Matplotlib: A 2D Graphics Environment. Comput. Sci. Eng. *9*, 90–95.

Konagurthu, A.S., Reboul, C.F., Schmidberger, J.W., Irving, J.A., Lesk, A.M., Stuckey, P.J., Whisstock, J.C., and Buckle, A.M. (2010). MUSTANG-MR Structural Sieving Server: Applications in Protein Structural Analysis and Crystallography. PLoS ONE *5*, e10048.

Kyte, J., and Doolittle, R.F. (1982). A simple method for displaying the hydropathic character of a protein. Journal of Molecular Biology *157*, 105–132.

McCoy, A.J., Grosse-Kunstleve, R.W., Adams, P.D., Winn, M.D., Storoni, L.C., and Read, R.J. (2007). Phaser crystallographic software. J Appl Crystallogr *40*, 658–674.

McPhillips, T.M., McPhillips, S.E., Chiu, H.-J., Cohen, A.E., Deacon, A.M., Ellis, P.J., Garman, E., Gonzalez, A., Sauter, N.K., Phizackerley, R.P., et al. (2002). Blu-Ice and the Distributed Control System: software for data acquisition and instrument control at macromolecular crystallography beamlines. J Synchrotron Radiat *9*, 401–406.

Miyamoto, S., and Kollman, P.A. (1992). SETTLE: an analytical version of the SHAKE and RATTLE algorithm for rigid water models. J. Comput. Chem. *13*, 952–962.

Ng, S.P., Billings, K.S., Ohashi, T., Allen, M.D., Best, R.B., Randles, L.G., Erickson, H.P., and Clarke, J. (2007). Designing an extracellular matrix protein with enhanced mechanical stability. Proc. Natl. Acad. Sci. U.S.a. *104*, 9633–9637.

Oostenbrink, C., Villa, A., Mark, A.E., and Van Gunsteren, W.F. (2004). A biomolecular force field based on the free enthalpy of hydration and solvation: The GROMOS force-field parameter sets 53A5 and 53A6. J. Comput. Chem. *25*, 1656–1676.

Tironi, I.G., Sperb, R., Smith, P.E., and van Gunsteren, W.F. (1995). A generalized reaction field method for molecular dynamics simulations. J. Chem. Phys. *102*, 5451.

Winn, M.D., Ballard, C.C., Cowtan, K.D., Dodson, E.J., Emsley, P., Evans, P.R., Keegan, R.M., Krissinel, E.B., Leslie, A.G.W., McCoy, A., et al. (2011). Overview of the CCP4 suite and current developments. Acta Crystallogr. D Biol. Crystallogr. *67*, 235–242.

**SI Data, Figures and Movies**

**Data S1.** Fasta file containing the sequence alignment used in consensus design of FN3con.

Data S2. Fasta file containing a sequence alignment of FN3con, Fibcon, FNfn10, Tencon and FNfn8.

**Figure S1.** Purification of FN3con. (A) SDS page gel of the FN3con purification process, showing cell lysis, NiNTA elution fraction and the size exclusion peaks from B. (B) Size exclusion chromatography plot of FN3con from NiNTA elution, with peak 3 being FN3con.

**Figure S2.** Reversible thermal folding of FN3con in 2 M GuHCl, monitored by CD at 222 nm. FN3con was heated from 20°C to 110°C (red) and cooled from 110°C to 20°C (blue). Respective non-linear fits were applied to the individual data points (*R^2^=0.98* forwards and *R^2^=0.98* reverse).

**Figure S3**. Plots of physiochemical properties from Tables 3 and 4 against determined melting temperatures of the FN3 domain. (A) Number of hydrogen bonds (solid line) and salt bridges (dashed line). (B) Solvent accessible surface area of respective FN3 domains plotted against temperature. (C) The grand average hydropathy (GRAVY) score of respective FN3 domains. (D) Solvent inaccessible cavity volume of FN3 domains in respect to their melting temperatures. Lower value indicated less cavity volume. (E) Mean protein packing value (OSP), larger value indicating better surface packing.

**Figure S4.** Approximate cavity volumes (red surface), including residues associated with the cavity volumes, as generated by the CASTp webserver using a 1.4 Å probe, for FN3con, Fibcon, FNfn10, Tencon, FNfn8 and TNfn3 (grey cartoon).


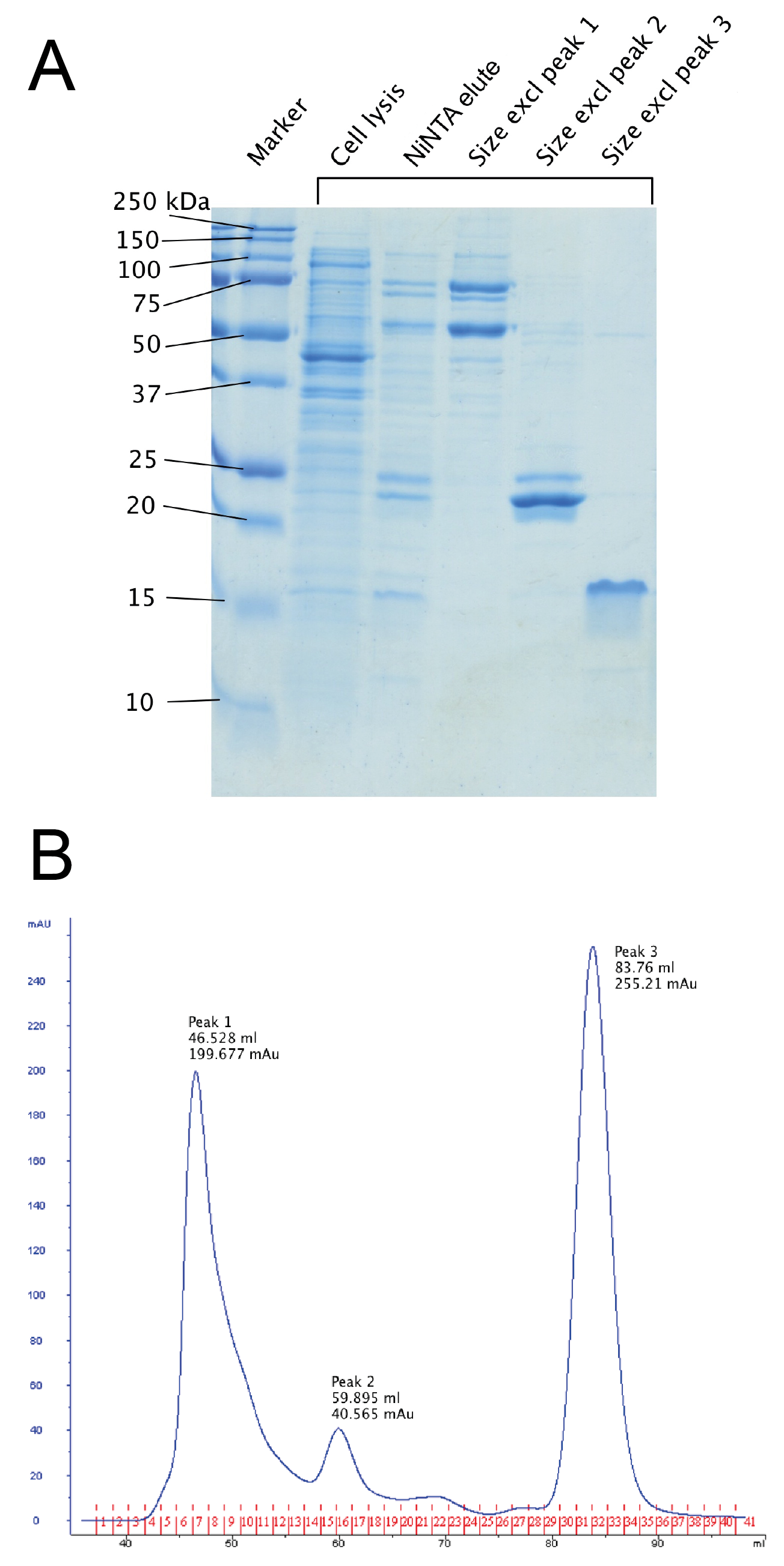


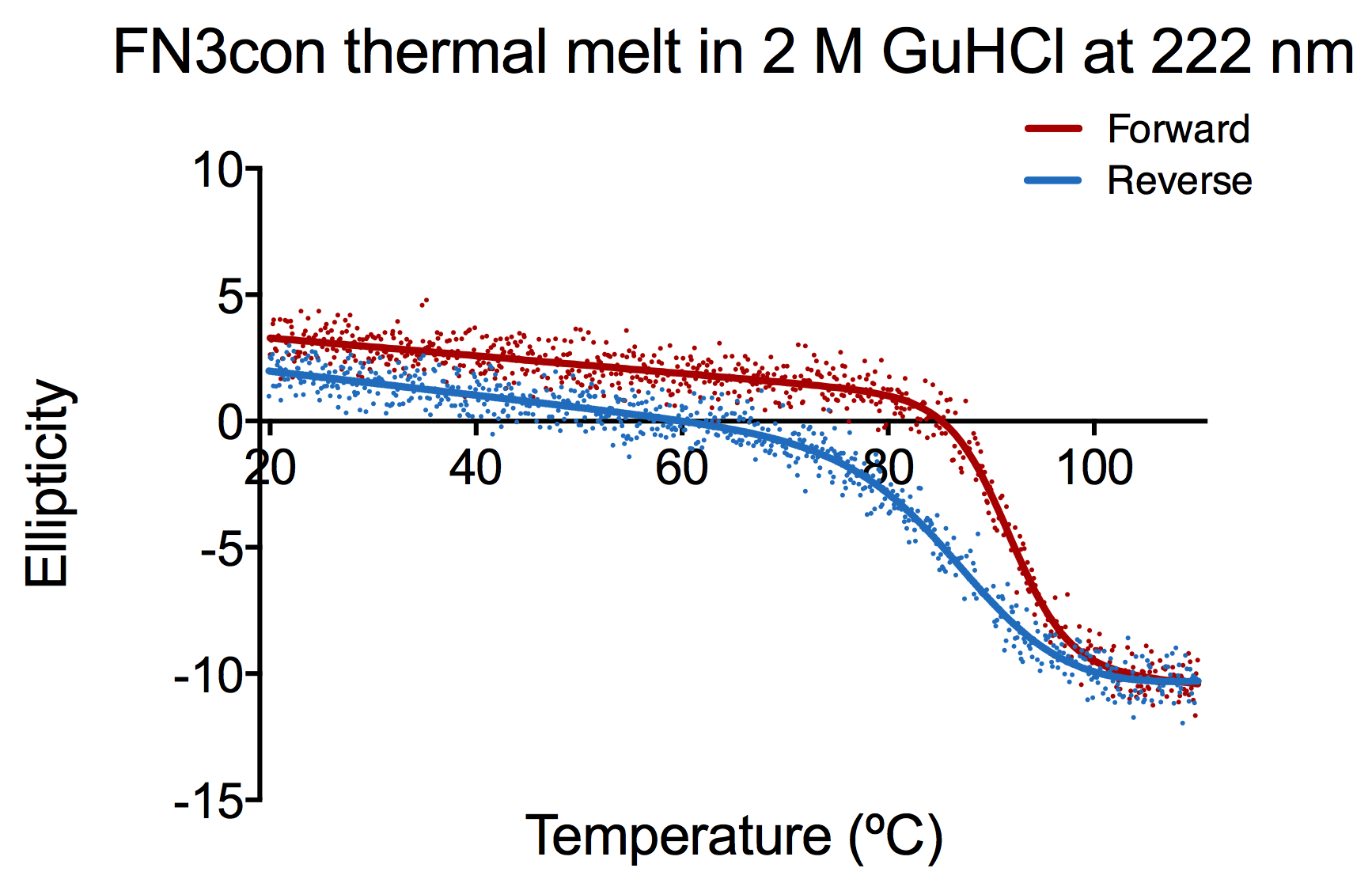


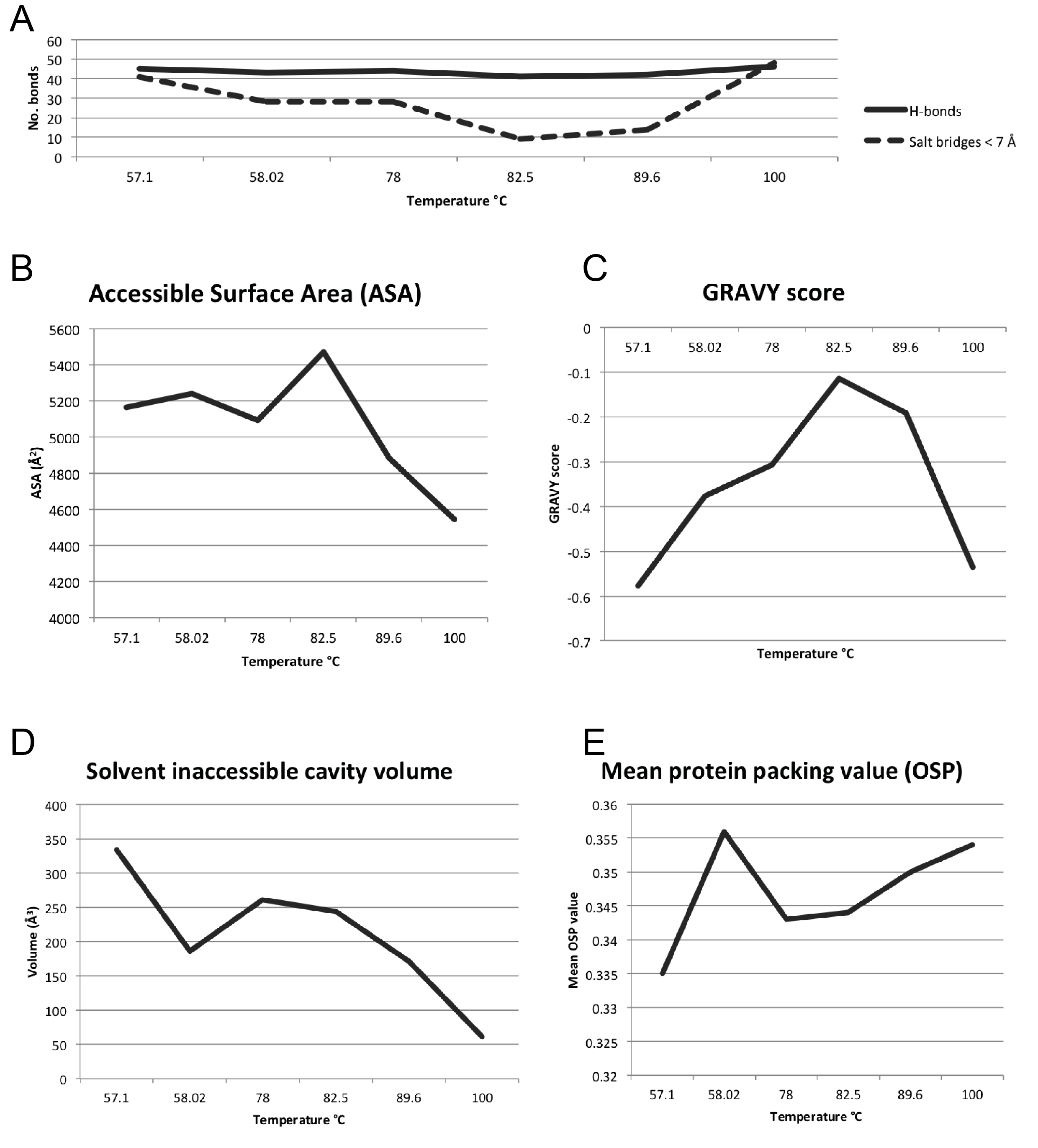


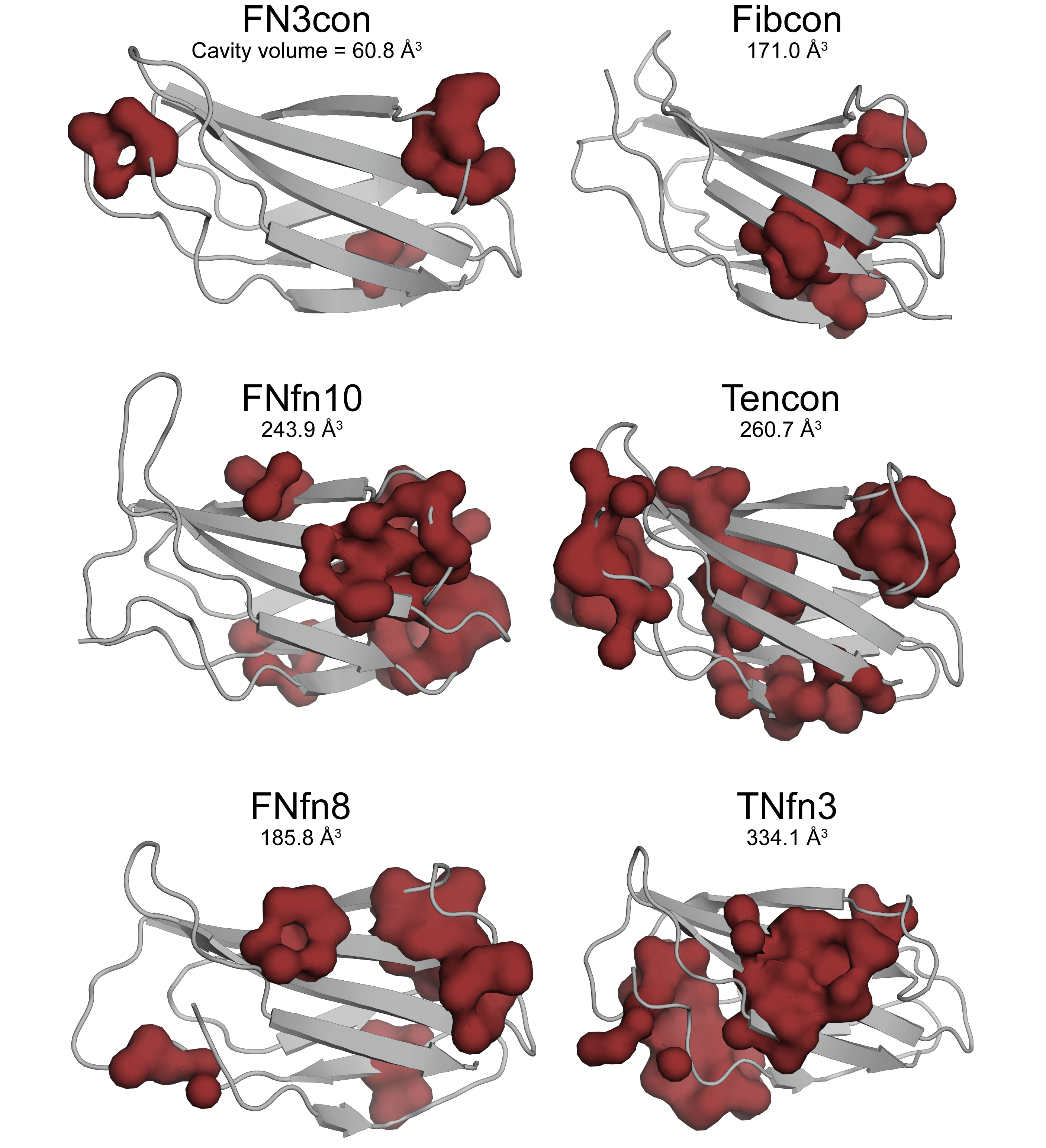


**Movie S1.** Molecular dynamics simulations of FN3con, Fibcon, FNfn10, Tencon, FNfn8 and TNfn3 at 368 K (94.85°C) for 2 μs, highlighting the dynamic alignment of the hydrophobic core. Structures are represented in cartoon form, with Cα atoms of hydrophobic residues represented as red spheres.

**Movie S2.** Molecular dynamics simulations of FN3con, Fibcon, FNfn10, Tencon, FNfn8 and TNfn3 at 368 K (94.85°C) for 2 μs, highlighting the positions of electrically charged residues.

**Movie S3.** Molecular dynamics simulations of FN3con, Fibcon, FNfn10, Tencon, FNfn8 and TNfn3 at 368 K (94.85°C) for 2 μs, highlighting the dynamical motions of tyrosine residues.
